# Effect of Deuteration on the Temperature Dependence of the Quadratic Electro-Optic Effect in KDP Crystals

**DOI:** 10.3390/ma18143290

**Published:** 2025-07-12

**Authors:** Marek Izdebski, Rafał Ledzion

**Affiliations:** Institute of Physics, Lodz University of Technology, Wólczańska 217/221, 93-005 Łódź, Poland; rafal.ledzion@p.lodz.pl

**Keywords:** KDP-type crystals, deuteration, quadratic electro-optic effect, intrinsic electro-optic coefficients

## Abstract

The results of precise measurements of the temperature dependencies of quadratic electro-optic coefficients, namely $g_{1111}-g_{1122}$ and $n_{o}^{3}g_{1111}-n_{e}^{3}g_{3311}$, in KH_2_PO_4_ (KDP) and KD_2_PO_4_ (DKDP) crystals at a wavelength of 632.8 nm are presented. We consider electro-optic coefficients describing changes in the optical impenetrability tensor resulting from an applied electric field, as well as intrinsic electro-optic coefficients defined in terms of induced polarization. The results show significant differences in the values of the analogous coefficients for the KDP and DKDP crystals and their temperature dependencies. Therefore, the quadratic electro-optic effect in KDP-type crystals cannot be easily described based solely on the contribution of PO_4_ tetrahedra, as assumed in current models of the linear effect. Moreover, the values of the intrinsic coefficients in the KDP and DKDP crystals differ even more than the corresponding usual electro-optic coefficients, which contradicts the conventional belief in their lower variability.

## 1. Introduction

Potassium Dihydrogen Phosphate (KDP) and Potassium Dideuterium Phosphate (DKDP) are commercial materials that are widely used in nonlinear optics. They are also excellent electro-optic crystals that are used in electro-optical modulators, Pockels cells, and Q-switches. The coefficients of the quadratic electro-optic effect are also of interest, because of their relations to quantities describing other phenomena, such as the second-order strain derivatives of electronic susceptibility [1] or the third-order susceptibility tensor [2]. In modulators that use the linear electro-optic effect, the quadratic electro-optic effect is an important source of harmonic distortions.

Traditionally, attempts to theoretically describe the linear electro-optic effect in KDP-type crystals have been based on the assumption that the contribution of K–O bonds can be neglected, owing to their high ionicity, and the contributions of H–O bonds cancel each other because of their isotropic distribution. Thus, the only significant contribution comes from P–O bonds [3,4]. These assumptions appeared to be confirmed by the good agreement between the theoretical values of the linear electro-optic coefficient $r_{63}$ predicted for KDP, DKDP, and ADP crystals and the experimental data [3]. However, the isotropic distribution of H–O bonds does not exclude their contribution to the quadratic electro-optic effect, and the theoretical analysis of this effect is much more complicated than had been assumed. Previous attempts to calculate the coefficients of the quadratic electro-optic effect in KDP and DKDP crystals used an extension of Bloembergen’s anharmonic oscillator model proposed by Kurtz and Robinson [5], and only allowed for a correct estimation of the order of magnitude [2]. To the best of our knowledge, no method for the precise prediction of the quadratic electro-optic coefficients of KDP-type crystals is currently available.

The structures of KDP and DKDP crystals, including the shape of PO_4_ tetrahedra, are almost the same [6,7], but the value of the crystal cell parameter *a* = *b* increases with an increasing deuterium content, while the parameter *c* remains unchanged. This effect can be explained by the fact that all H–O and D–O bonds are perpendicular to the *c*-axis (i.e., *Z*-axis) and the O–H–O bond length at room temperature is 2.487 Å, while the O–D–O bond length is 2.519 Å [8]. Hence, the experimental data on the quadratic electro-optic effect obtained with an electric field applied along the *X* or *Y* axis should reveal differences between the contributions of the H–O and D–O bonds, whereas a field applied along the *Z* axis should not induce a clear contribution from hydrogen bonds.

Several experimental attempts have been made to investigate the effect of deuteration on the quadratic electro-optic effect in KDP-type crystals; however, these experiments have usually been limited to room temperature. Unfortunately, according to the results presented in this work, the often-considered effective quadratic electro-optic coefficient $g_{1111}-g_{1122}$ takes very similar values in KDP and DKDP crystals at room temperature, while at other temperatures the differences may be larger. Moreover, the measurement methods used previously do not appear to provide sufficient accuracy, which has led to the conclusion that the quadratic electro-optic coefficients in KDP and DKDP crystals have comparable values within experimental uncertainties [1,9,10,11].

There are very few previous studies on the temperature dependence of the quadratic electro-optic effect in KDP and DKPD crystals in the paraelectric phase at temperatures well above the ferroelectric–paraelectric phase transition temperature. The temperature dependencies of the $g_{1111}-g_{1122}$ and $n_{o}^{3}g_{1111}-n_{e}^{3}g_{3311}$ coefficients in DKDP crystals were presented in [11]; however, because of a lack of data, they could not be compared with the analogous dependencies for the KDP crystal. To the best of our knowledge, the present study is the first to report the temperature dependence of the $n_{o}^{3}g_{1111}-n_{e}^{3}g_{3311}$ coefficient in the KDP crystal. The temperature dependence of the $g_{1111}-g_{1122}$ coefficient in the KDP crystal was recently presented in [12], but the differences between the results for the KDP and DKDP crystals have not been discussed previously. Moreover, significant differences in the measurement methods used in [11,12] and their accuracies make it difficult to draw any unambiguous conclusions.

The aim of this study was to investigate and compare the temperature dependencies of the quadratic electro-optic coefficients $g_{1111}-g_{1122}$ and $n_{o}^{3}g_{1111}-n_{e}^{3}g_{3311}$ in KDP and DKDP crystals using the precise measurement method described in our earlier work [12], which allows for an accuracy of approximately 1.0 to 1.5%. As we used the same apparatus, method, and measurement procedure for both KDP and DKDP crystals, we could fully exploit the resolution of our measurements to show the differences between the crystal properties. To the best of our knowledge, this is the first comparison that does not depend on experimental data taken from other studies employing different approaches.

In this study, we define the electro-optic coefficients in a typical manner, as follows [13,14,15]:

(1) $$B_{\mathrm{ij}}\left(E\right)-B_{\mathrm{ij}}\left(0\right)=r_{\mathrm{ijk}}E_{k}+g_{\mathrm{ijkl}}E_{k}E_{l}+\cdots ,$$

where $B_{\mathrm{ij}}$ are the components of the optical impermeability tensor, **E** is the applied low-frequency electric field, and $r_{\mathrm{ijk}}$ and $g_{\mathrm{ijkl}}$ are the coefficients of the linear and quadratic electro-optic effects, respectively. Following the approach suggested by Pockels, we also considered the intrinsic electro-optic coefficients defined in terms of polarization **P** rather than the applied field [13,16,17,18]:

(2) $$B_{\mathrm{ij}}\left(P\right)-B_{\mathrm{ij}}\left(0\right)=l_{\mathrm{ijk}}P_{k}+f_{\mathrm{ijkl}}P_{k}P_{l}+\cdots ,$$

which are considered in the literature to be less dependent on the material and temperature than the corresponding $r_{\mathrm{ijk}}$ and $g_{\mathrm{ijkl}}$ coefficients. However, in our opinion, this hypothesis is well supported by experimental data only for the linear electro-optic effect; for the quadratic effect, more intensive and precise measurements are still needed. The new results presented in this work show that the intrinsic quadratic electro-optic coefficients may be even more variable than the $g_{\mathrm{ijkl}}$ coefficients.

To determine the intrinsic coefficients $f_{\mathrm{ij}11}$, it is necessary to know the values of the corresponding $g_{\mathrm{ij}11}$ coefficients and the relative permittivity $\varepsilon_{11}$. Nevertheless, the values of $\varepsilon_{11}$ reported in various studies at room temperature differ significantly, and their temperature dependencies are unknown for DKDP crystals. Therefore, this work also includes measurements of the temperature dependence of $\varepsilon_{11}$ performed for the same KDP and DKDP crystals that were used in our electro-optical measurements.

## 2. Materials and Methods

Our measurements were performed on two crystals cut in the form of parallelepipeds manufactured by Cobrabid-Optica Warsaw, Poland. The dimensions of the DKDP crystal, 4.250 × 25.112 × 25.379 mm^3^ (*X* × *Y* × *Z*), were measured using a Mitutoyo micrometer model MDC-25PX (Mitutoyo, Kanagawa, Japan) with an accuracy of 1 μm. In the case of the KDP crystal of dimensions 6.238 × 29.24 × 38.75 mm^3^ (*X* × *Y* × *Z*), the thickness in the *X*-axis direction was measured with a Mitutoyo micrometer, while the other dimensions were measured using a Mitutoyo digital caliper model CD-6 ASX with an accuracy of 0.01 mm.

Because KDP-type crystals absorb moisture [19], the both crystals were dehydrated using acetone and heating. The heat treatment was also applied to improve the optical properties of KDP and DKDP crystals [20]. The electrodes were then painted with silver conductive paint on the faces perpendicular to the *X* axis. Each crystal was placed in a glass spectrophotometric cuvette and flooded with Polsil OM-500 methyl silicone oil (viscosity 500 cSt at 25 °C) manufactured by Silikony Polskie Ltd., Nowa Sarzyna, Poland, which protected the hygroscopic crystals from moisture and improved heat transfer. The similar values of the refractive index in the oil and crystals also allow for the effective suppression of multiple reflections and the scattering of the light beam on the crystal faces. Because of the extremely weak quadratic electro-optic effect $g_{1111}-g_{1122}\approx 4\times 10^{-22}$ ${m^{2}V}^{-2}$ and low relative permittivity ε≈2.7 in methyl silicone oil [21], the electric field dispersed in the oil around the electrodes on the crystal should not contribute significantly to the electro-optical and dielectric measurements of the crystals.

Each crystal rested freely on the bottom of the cuvette and its position was stabilized with thin Teflon strips, which did not restrict crystal deformation in the applied electric field. Because the frequency of the applied field was far below the piezoresonance frequency, the results correspond to unclamped crystals.

Electro-optical measurements of each crystal were performed for the following two configurations of the incident light direction **σ** and the applied electric field **E**:

**σ** = (0, 0, 1),  **E** = (*E*, 0, 0), (3)

**σ** = (0, 1, 0),  **E** = (*E*, 0, 0). (4)

As it results from the forms of linear and quadratic electro-optic tensors for the $\bar{4}2m$ symmetry [13,14,15], the linear electro-optic effect is suppressed in both configurations, and the polarimetric method allows for the measurement of the $n_{o}^{3}\left(g_{1111}-g_{1122}\right)$ effective quadratic electro-optic coefficient in configuration (3) and $n_{o}^{3}g_{1111}-n_{e}^{3}g_{3311}$ in configuration (4), where $n_{o}$ and $n_{e}$ are the ordinary and extraordinary refractive indices, respectively.

The polarimetric method for electro-optical measurements has several varieties that differ in the type of modulating waveform, orientation of elements, operating point of the electro-optical modulator on its transmission characteristics, method of controlling this point, and measurement procedure. All of this results in large differences in the achieved accuracies and duration of measurements. Because achieving the best possible measurement accuracy was a priority in this study, we used the method described in detail in [12]. In this method, measurements are performed at multiple operating points on the transmission characteristic of an electro-optical modulator as a function of the total phase retardation, instead of at only one specific point, as in the traditional approach. Because only the relative positions of the operating points must be controlled, this can be achieved easily and precisely using a stepper motor to rotate the analyzer in a Sénarmont-type setup. High accuracy also results from the use of an improved mathematical model of the measurement system, which includes the partial interference of two waves passing through the sample, the inaccuracy of the quarter-wave plate used, and possible dichroism in the sample and quarter-wave plate. The equipment and measurement procedure used in this work are the same as those described in detail in [12], except for the use of a different laser (Lasos LGK 7665 P with a power of 15 mW and a wavelength of 632.8 nm) and immersion oil (Polsil OM-500 with a ten times higher viscosity than in the previous study).

In the case of the KDP crystal, measurements in both configurations (3) and (4) were performed at temperatures increasing from 20 °C to 80 °C in steps of 5 °C. Because the electrical conductivity of the DKDP crystal increased faster with increasing temperature than that of the KDP, we limited the maximum temperature of this crystal to 60 °C. Measurements at higher temperatures can destroy the crystal, because of the heat generated by the current flowing through the crystal. To ensure that the results were independent of the amplitude of the applied electric field, measurements at each temperature were performed for 14 levels of sinusoidally varying voltages from approximately 720 to 2030 V RMS, and the results were averaged as described previously in [12]. The measurements required approximately 24 h for each temperature, including the total time of temperature stabilization and the duration of the measurements. Because this time must be multiplied by the number of temperatures, by configurations (3) and (4), and by two crystals, it was impossible for us to perform measurements for many combinations of temperatures and frequencies of the applied modulating field. Therefore, we chose a single frequency of 417 Hz for all the measurements. The selected frequency is a compromise between the noise level in the detection path, which decreases with increasing frequency, and the loading of the high-voltage transformer by the capacitive load, which increases with increasing frequency. It is also important that the selected frequency differs from all harmonics of the 50 Hz supply. Before the actual measurements, we verified that our electro-optical measurements did not depend significantly on the frequency in the band from 217 Hz to 1017 Hz at room temperature. Higher frequencies were not available to us due to the limitations of the high-voltage transformer used. Although simpler and faster measurement procedures are available, the simplification of the method results in a reduction in accuracy, which is disadvantageous for this study.

To calculate the values of the intrinsic electro-optic coefficients $f_{1111}-f_{1122}$ and $n_{o\;}^{3}f_{1111}-n_{e\;}^{3}f_{3311}$, the values of the relative permittivity $\varepsilon_{11}$ must be known. As is known from the literature, the temperature dependence of $\varepsilon_{11}$ in KDP crystals obeys the Curie–Weiss law (except for minor deviations at low temperatures, which are insignificant here). The values of the constants occurring in this law are given in [22]. Unfortunately, the temperature dependence for DKDP crystals at temperatures above room temperature has not been established. Moreover, the $\varepsilon_{11}$ values known at room temperature show significant discrepancies from 42 [23] to 46.7 [22] for KDP crystals and from 50 [23] to 65.0 [24] for DKDP crystals. The large discrepancy in reported $\varepsilon_{11}$ values may be due to several reasons, such as frequency dependence, different crystals quality (dependent on growth method, growth rate and impurities), and measurements made for unclamped or clamped crystals. Moreover, in the case of KDP-type crystals, which absorb moisture [19], we observed significant differences between the crystal dehydrated before measurements (as in this work) and the non-dehydrated crystal, but a detailed description of all the mentioned measurement conditions is usually not given in the literature. Therefore, our calculations of the intrinsic electro-optic coefficients were based on our own $\varepsilon_{11}$ measurements made for the same crystals and electrodes used in the electro-optical measurements. The relative permittivity was calculated as $\varepsilon_{11}=C_{p}d/\varepsilon_{0}S$, where $C_{p}$ is the capacitance measured using the LCR meter GW INSTEK model LCR-6100 with a parallel equivalent circuit model of capacitance and resistance, *d* is the crystal thickness measured in the *X*-axis direction, *S* is the surface area of the crystal, and $\varepsilon_{0}$ is the vacuum permittivity. The measurements were performed at temperatures increasing from 18 °C to 80 °C for the KDP crystal and from 18 °C to 60 °C for the DKDP crystal in steps of 2 °C.

## 3. Results

### 3.1. Quadratic Electro-Optic Coefficients

**Figure 1:**
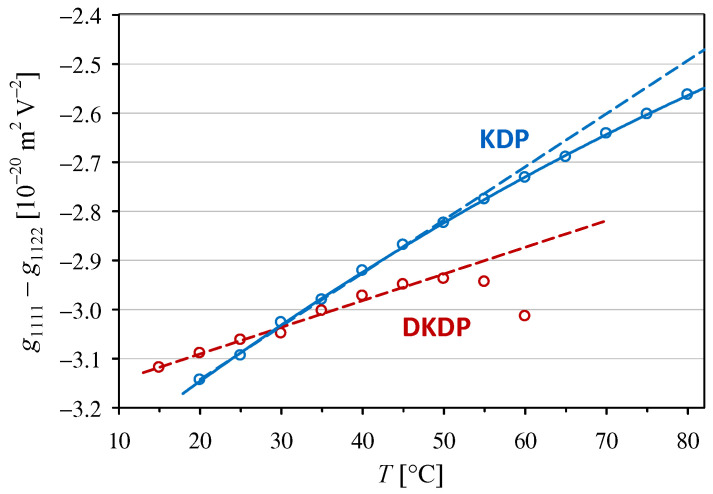
Temperature dependencies of the quadratic electro-optic coefficient $g_{1111}-g_{1122}$ in KDP and DKDP crystals. The dashed lines indicate linear trends with the parameters given in Table 1. The full line shows second-order polynomial interpolation for KDP $g_{1111}-g_{1122}\;\left[10^{-22}\;m^{2}V^{-2}\right]=-3.396+0.01320T-0.0000350T^{2}$ for temperature *T* given in [°C].

Configuration (3) allows for the direct measurement of the effective electro-optic coefficient $n_{o}^{3}\left(g_{1111}-g_{1122}\right)$. To obtain the temperature dependencies of the $g_{1111}-g_{1122}$ coefficient, which are shown in Figure 1, we used the temperature dependencies of $n_{o}$ in KDP and DKDP crystals given by Ghosh and Bhar [25]. It is worth noting that the values of $g_{1111}-g_{1122}=(-3.09\;\pm \;0.04)\times 10^{-20}$ and $(-3.06\;\pm \;0.04)\times 10^{-20}$ $m^{2}V^{-2}$ obtained at room temperature (25 °C) for the KDP and DKDP, respectively, are comparable within the experimental uncertainty, which confirms the conclusions drawn from earlier experimental data for room temperature formulated in [9]. However, our new results show that KDP and DKDP crystals clearly differ in the temperature dependence of the $g_{1111}-g_{1122}$ coefficient. If the temperature range is limited to ≤50 °C, where the dependencies can be approximated by a linear trend (dashed lines in Figure 1), the temperature coefficient for KDP turns out to be almost twice as large in absolute value than that for the DKDP crystal (see $a_{g}$ coefficient in Table 1).

**Figure 2:**
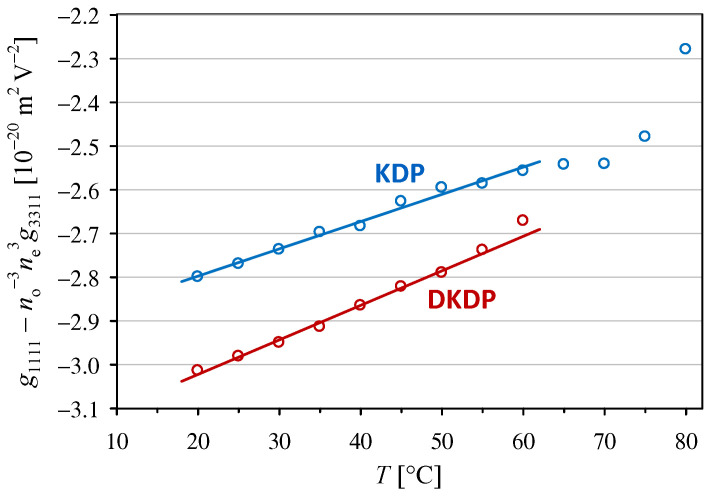
Temperature dependencies of the quadratic electro-optic coefficient $g_{1111}-n_{o}^{-3}n_{e}^{3}g_{3311}$ in KDP and DKDP crystals. The full lines indicate linear trends with the parameters given in Table 1.

In the case of the results obtained in configuration (4), we cannot completely decouple the temperature dependence of the $n_{o}^{3}g_{1111}-n_{e}^{3}g_{3311}$ coefficient from the contribution of the temperature dependence of the refractive indices. To reduce this contribution to a negligible level, we considered the effective coefficient $g_{1111}-n_{o}^{-3}n_{e}^{3}g_{3311}$, where, according to earlier interferometric measurements of the KDP crystal, $g_{1111}$ is almost five times larger in absolute value than $g_{3311}$ [26] and the relative changes in $n_{o}^{-3}n_{e}^{3}$ are in the order of 1 per mille in the temperature range from 20 °C to 80 °C [25]. As can be seen in Figure 2, the temperature dependencies of $g_{1111}-n_{o}^{-3}n_{e}^{3}g_{3311}$ for both crystals are close to linear at temperatures below 60 °C. These dependencies differ from those shown in Figure 1 because the values of $g_{1111}-n_{o}^{-3}n_{e}^{3}g_{3311}$ obtained for the DKDP crystal are always significantly higher in absolute value than those for KDP, whereas the temperature coefficient $a_{g}$ assumes similar values for both crystals (see Table 1).

**Figure 3:**
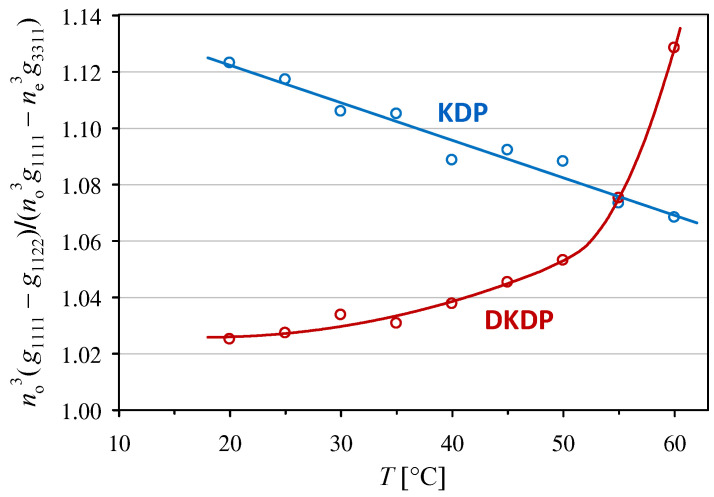
Temperature dependence of the ratio of the effective quadratic electro-optic coefficient $g_{1111}-g_{1122}$ to $g_{1111}-n_{o}^{-3}n_{e}^{3}g_{3311}$ in KDP and DKDP crystals.

The differences in the temperature dependencies of the $g_{1111}-g_{1122}$ and $g_{1111}-n_{o}^{-3}n_{e}^{3}g_{3311}$ coefficients are clearly visible in Figure 3, where the ratio of these coefficients is plotted against the temperature. The ratio decreases with increasing temperature for the KDP crystal, whereas the dependence increases for the DKDP crystal.

### 3.2. Dielectric Properties of Crystals

**Figure 4:**
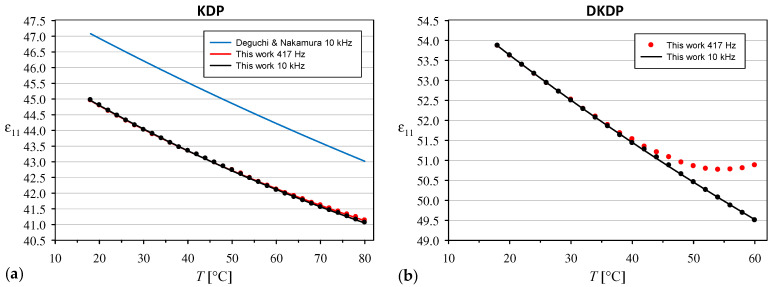
Temperature dependencies of the relative permittivity $\varepsilon_{11}$ for (**a**) KDP and (**b**) DKDP crystals. The full lines are interpolations with the Curie–Weiss formula (5).

The temperature dependence of the relative permittivity $\varepsilon_{11}$ for the KDP crystal is shown in Figure 4a. The results obtained at frequencies of 417 Hz and 10 kHz were almost identical at room temperature, whereas a slight difference was observed with increasing temperature, reaching up to 0.072 at 80 °C. Our $\varepsilon_{11}$ values are approximately 5% lower than the results reported for KDP crystals at 10 kHz by Deguchi and Nakuamura [22], but the relative changes with increasing temperature are very similar. On the other hand, our value of 44.4 at 25 °C is slightly higher than some earlier results for room temperature, such as 43.2 for the unclamped KDP crystal at 25 °C given in [24].

In the case of the DKDP crystal, the effect of frequency on the $\varepsilon_{11}$ value increased much faster with increasing temperature and the difference between the results obtained at 417 Hz and 10 kHz reached 1.37 at the highest temperature of 60 °C (Figure 4b). Therefore, it was necessary to consider which series of $\varepsilon_{11}$ measurements should be used to calculate the intrinsic electro-optic coefficients, based on the usual electro-optic coefficients obtained at 417 Hz. Considering that $\varepsilon_{11}$ increases with decreasing frequency and the intensity of this effect clearly correlates with the electrical conductivity of the crystal, which increases rapidly with the increasing temperature of the DKDP crystal, we observed the migration of electric charges accumulated in the bulk of a real crystal containing defects [23]. Because the electro-optic effect results from field-induced changes in the properties of the crystal itself rather than from charge migration through the crystal, in further calculations we used only the values of $\varepsilon_{11}$ obtained at a frequency of 10 kHz, at which the experimental temperature dependencies of $\varepsilon_{11}$ fit very well the Curie–Weiss law in the following form [22]:

(5) $$\varepsilon_{11}=\varepsilon_{11}^{\infty }+\frac{C_{11}}{T-T_{11}},$$

where $\varepsilon_{11}^{\infty }$ is high-frequency relative permittivity. In the case of the KDP crystal, the best fit was obtained for $\varepsilon_{11}^{\infty }=26.40$, $C_{11}=4314\;K$, and $T_{11}=58.55\;K$, with the mean absolute error (*MAE*) between the values predicted from Equation (5) and the experimental values of 0.013. The analogous results obtained for the DKDP crystal are $\varepsilon_{11}^{\infty }=18.40$, $C_{11}=10661\;K$, $T_{11}=-9.49\;K$, and *MAE* = 0.010.

### 3.3. Intrinsic Quadratic Electro-Optic Coefficients

**Figure 5:**
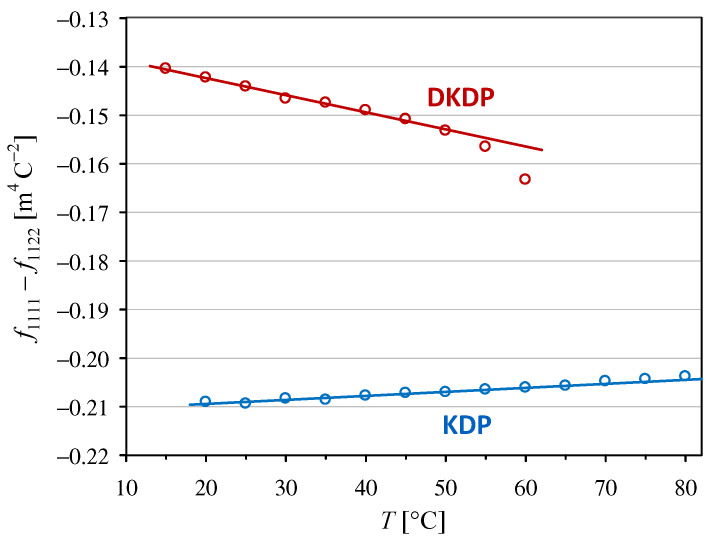
Temperature dependencies of the intrinsic quadratic electro-optic coefficient $f_{1111}-f_{1122}$ in KDP and DKDP crystals. The full lines indicate linear trends with the parameters given in Table 2.

**Figure 6:**
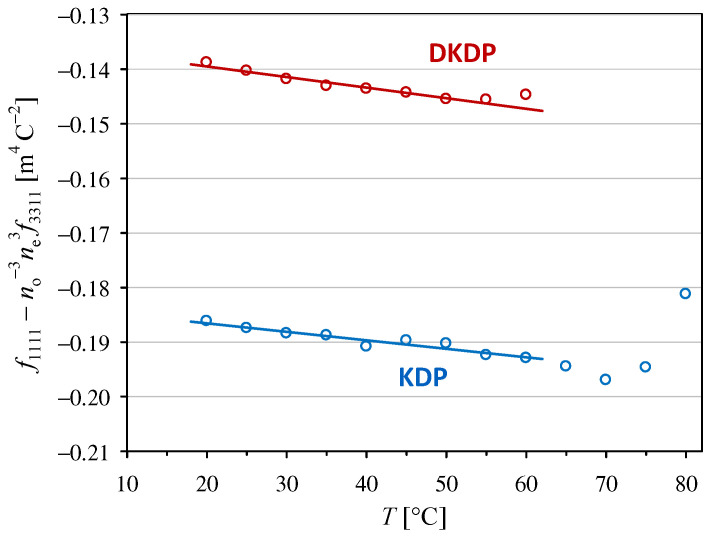
Temperature dependencies of the intrinsic quadratic electro-optic coefficient $f_{1111}-n_{o}^{-3}n_{e\;}^{3}f_{3311}$ in KDP and DKDP crystals. The full lines indicate linear trends with the parameters given in Table 2.

The intrinsic quadratic electro-optic coefficients $f_{\mathrm{ijkl}}$ defined in terms of polarization can be calculated as follows:

(6) $$f_{\mathrm{ijkl}}=\frac{g_{\mathrm{ijkl}}}{\varepsilon_{0}^{2}\left(\varepsilon_{\mathrm{kk}}-1\right)\left(\varepsilon_{\mathrm{ll}}-1\right)},$$

where $\varepsilon_{0}$ is the vacuum permittivity and $\varepsilon_{\mathrm{kk}}$ and $\varepsilon_{\mathrm{ll}}$ are the principal values of the relative permittivities for a low-frequency modulating field. Hence, using our experimental results presented in Section 3.1 and Section 3.2, we calculated the effective intrinsic quadratic electro-optic coefficients, namely $f_{1111}-f_{1122}$ shown in Figure 5 and $f_{1111}-n_{o}^{-3}n_{e\;}^{3}f_{3311}$ shown in Figure 6. The parameters for the linear trends in these plots are listed in Table 2.

The values of $f_{1111}-f_{1122}$ obtained in this work for the KDP crystal are slightly higher than our previous values given in [12], which is mostly owing to the use of our $\varepsilon_{11}$ measurements instead of the values from [22]. However, this change did not significantly affect the value of the temperature coefficient $a_{f}$ related to $f_{1111}-f_{1122}$ for KDP. To the best of our knowledge, the other three temperature dependencies presented in Figure 5 and Figure 6 have not been reported previously.

## 4. Discussion

The values of the $g_{1111}-g_{1122}$ and ${n_{o}^{3}g}_{1111}-n_{e}^{3}g_{3311}$ coefficients in KDP and DKDP crystals have been reported in several studies, but in most cases only at room temperature (see Table 3). Unfortunately, at room temperature, the values of $g_{1111}-g_{1122}$ in the KDP and DKDP crystals are very close and their difference is below the measurement accuracy. However, the results presented in this work show that the temperature dependence of the $g_{1111}-g_{1122}$ coefficient is much stronger for the KDP crystal than for the DKDP crystal.

It is worth noting that the results obtained for the coefficient $g_{1111}-n_{o}^{-3}n_{e}^{3}g_{3311}$ are qualitatively different from those for $g_{1111}-g_{1122}$. We observed that the temperature dependencies of $g_{1111}-n_{o}^{-3}n_{e}^{3}g_{3311}$ in KDP and DKDP crystals differed only slightly, but the absolute values of this coefficient were higher in the DKDP crystal over the entire temperature range from 20 °C to 60 °C. At room temperature (25 °C), the difference is 0.21 (0.65 for the coefficient ${n_{o}^{3}g}_{1111}-n_{e}^{3}g_{3311}$ considered in earlier works), which corresponds to about 6.5% of the measured value. The observation of such a difference was possible because of the use of our new precise measurement method described in [12], whereas previous methods only allowed us to state that the values are comparable within experimental inaccuracies.

Intrinsic electro-optic coefficients are traditionally considered to be less dependent on the material and temperature than the coefficients defined in terms of an applied electric field. However, our experimental results obtained at room temperature (25 °C) show that the absolute value of the intrinsic coefficient $f_{1111}-f_{1122}$ in the DKDP crystal is 31% lower than that in the KDP crystal, while the values of $g_{1111}-g_{1122}$ are almost equal in both crystals (compare Figure 1 and Figure 5). Similarly, at room temperature, the absolute value of the intrinsic coefficient $f_{1111}-n_{o}^{-3}n_{e\;}^{3}f_{3311}$ in the DKDP crystal differs by 25% from the value in the KDP crystal, but the difference is only 8% when the coefficient $g_{1111}-n_{o}^{-3}n_{e}^{3}g_{3311}$ is considered (compare Figure 2 and Figure 6).

A comparison of the temperature dependencies of the intrinsic electro-optic coefficients and the coefficients defined in terms of an applied field did not yield unambiguous results. The temperature dependence of the intrinsic coefficient $f_{1111}-f_{1122}$ was stronger than that of the $g_{1111}-g_{1122}$ coefficient in the DKDP crystal, but the results obtained for the KDP crystal lead to the opposite conclusion. The intrinsic coefficient $f_{1111}-n_{o}^{-3}n_{e\;}^{3}f_{3311}$ was less temperature dependent than $g_{1111}-n_{o}^{-3}n_{e}^{3}g_{3311}$ for both crystals (see Table 1 and Table 2).

## 5. Conclusions

In this study, we measured the temperature dependencies of the quadratic electro-optic coefficients $g_{1111}-g_{1122}$ and $g_{1111}-n_{o}^{-3}n_{e}^{3}g_{3311}$ in KDP and DKDP crystals. The results show significant differences between the values of the $g_{1111}-n_{o}^{-3}n_{e}^{3}g_{3311}$ coefficient in KDP and DKDP crystals, ranging from 8% to 4% for temperatures ranging from 20 °C to 60 °C. In the case of the $g_{1111}-g_{1122}$ coefficient, the values obtained for the KDP and DKDP crystals were very similar at room temperature, but the temperature dependence in the KDP crystal was almost twice as large as that in DKDP. These results represent a significant improvement over the results of previous imprecise and fragmentary measurements, which only led to the conclusion that the results were comparable within experimental uncertainties.

We have also shown that deuteration has an even stronger effect on the values of the intrinsic electro-optic coefficients, which are defined in terms of polarization. This result contradicts the traditional belief that the intrinsic coefficients are less material-dependent than the coefficients defined in terms of an applied electric field.

Because the structures of KDP and DKDP crystals are almost the same, the observed deuteration effect implies that H–O and D–O bonds make significant and different contributions to the quadratic electro-optic effect. To the best of our knowledge, there is currently no theoretical model that allows the quantitative prediction of this contribution. The model of the linear electro-optic effect in KDP family crystals proposed by Shih and Yariv [3], which uses the isotropic distribution of hydrogen bonds to exclude their contribution to the phenomena described by odd-order tensors, cannot easily be extended to the quadratic effect.

## Figures and Tables

**Table 1:** Parameters of the linear trends $g_{\mathrm{eff}}=g_{0}(1+a_{g}T)$ shown in Figure 1 and Figure 2 for *T* given in [°C].

Crystal	Effective Coefficient	$g_{0}$ [10^−20^ m^2^V^−2^]	$a_{g}$ [°C^−1^]	Temp. Range [°C]
KDP	$g_{1111}-g_{1122}$	−3.3588(78) *	−0.003223(64)	20~50
DKDP	$g_{1111}-g_{1122}$	−3.1993(89) *	−0.001698(81)	15~50
KDP	$g_{1111}-n_{o}^{-3}n_{e}^{3}g_{3311}$	−2.922(11) *	−0.002135(92)	20~60
DKDP	$g_{1111}-n_{o}^{-3}n_{e}^{3}g_{3311}$	−3.1805(85) *	−0.002484(69)	20~55

* the values of the standard deviation of the *g*_0_ coefficient given in brackets describe only the statistical dispersion of the experimental data processed using the least squares method. The maximum uncertainty of *g*_0_ resulting from the accuracy of the measurement method is 1% for the $g_{1111}-g_{1122}$ coefficient and 1.5% for the $g_{1111}-n_{o}^{-3}n_{e}^{3}g_{3311}$ coefficient.

**Table 2:** Parameters of the linear trends $f_{\mathrm{eff}}=f_{0}(1+a_{f}T)$ shown in Figure 4 and Figure 5 for *T* given in [°C].

Crystal	Effective Coefficient	$f_{0}$ [m^4^C^−2^]	$a_{f}$ [°C^−1^]	Temp. Range [°C]
KDP	$f_{1111}-f_{1122}$	−0.21109(30) *	−0.000390(34)	20~60
DKDP	$f_{1111}-f_{1122}$	−0.13531(43) *	+0.002600(92)	15~50
KDP	$f_{1111}-n_{o}^{-3}n_{e\;}^{3}f_{3311}$	−0.18347(74) *	+0.00085(10)	20~60
DKDP	$f_{1111}-n_{o}^{-3}n_{e\;}^{3}f_{3311}$	−0.13560(64) *	+0.00143(12)	20~55

* the values of the standard deviation of the *f*_0_ coefficient given in brackets describe only the statistical dispersion of the experimental data processed using the least squares method. The maximum uncertainty of *f*_0_ resulting from the accuracy of the measurement method is 1.1% for the $f_{1111}-f_{1122}$ coefficient and 1.6% for the $f_{1111}-n_{o}^{-3}n_{e\;}^{3}f_{3311}$ coefficient.

**Table 3:** Comparison of experimental values of the quadratic electro-optic coefficients at room temperature for a wavelength of 632.8 nm in KDP and DKDP crystals.

Crystal	Effective Coefficient	$g_{\mathrm{eff}}$ [10^−20^ m^2^V^−2^]	Ref.	*T* [°C]	*f* [Hz]
KDP	$g_{1111}-g_{1122}$	−3.09 ± 0.04	this work	25	417
KDP	$g_{1111}-g_{1122}$	−3.07 ± 0.03	[12]	25	417
KDP	$g_{1111}-g_{1122}$	−3.2 ± 0.2	[26]	21	391
KDP	$g_{1111}-g_{1122}$	−4.2 ± 0.6	[27]	no data	400
KDP	$\left|g_{1111}-g_{1122}\right|$	2.5 ± 0.5	[28]	no data	180~390
KDP	$n_{o}^{3}g_{1111}-n_{e}^{3}g_{3311}$	−9.49 ± 0.15	this work	25	417
KDP	$n_{o}^{3}g_{1111}-n_{e}^{3}g_{3311}$	−9.5 ± 2.0	[26]	21	391
KDP	$\left|n_{o}^{3}g_{1111}-n_{e}^{3}g_{3311}\right|$	9.3 ± 0.6	[29]	no data	no data
DKDP	$g_{1111}-g_{1122}$	−3.06 ± 0.04	this work	25	417
DKDP	$\left|g_{1111}-g_{1122}\right|$	2.82 ± 0.06 *	[11]	22	417
DKDP	$\left|g_{1111}-g_{1122}\right|$	3.40 ± 0.25	[10]	21	80~400
DKDP	$n_{o}^{3}g_{1111}-n_{e}^{3}g_{3311}$	−10.14 ± 0.15	this work	25	417
DKDP	$\left|n_{o}^{3}g_{1111}-n_{e}^{3}g_{3311}\right|$	10.5 ± 0.3	[11]	22	417
DKDP	$\left|n_{o}^{3}g_{1111}-n_{e}^{3}g_{3311}\right|$	10.0 ± 0.6 10.4 ± 0.5 ^†^	[9]	19~23	300~420

*—calculated based on $n_{o}^{3}\left|g_{1111}-g_{1122}\right|=(9.6\;\pm \;0.2)\times 10^{-20}\;m^{2}V^{-2}$ [11] and $n_{o}=1.50421$ [25], ^†^—results obtained for two measured crystals.

## Data Availability

The original contributions presented in this study are included in the article. Further inquiries can be directed to the corresponding author.
